# HERV Dysregulation in a Case of Myalgic Encephalomyelitis and Multiple Sclerosis Responsive to Rituximab

**DOI:** 10.3390/ijms26104885

**Published:** 2025-05-20

**Authors:** Eva Martín-Martínez, Sara Gil-Perotin, Karen Giménez-Orenga, Lucas Barea-Moya, Elisa Oltra

**Affiliations:** 1 National Health Service, Manises Hospital, 46940 Valencia, Spain; evamartinmartinez@gmail.com; 2 Neurology, Hospital Universitario y Politécnico La Fe, 46026 Valencia, Spain; sara.garcia@uv.es; 3 Multiple Sclerosis Unit, Neurology, Hospital Universitario y Politécnico La Fe, 46026 Valencia, Spain; 4 CIBER, Instituto de Salud Carlos III, 28029 Madrid, Spain; 5 Escuela de Doctorado, Universidad Católica de Valencia San Vicente Mártir, 46001 Valencia, Spain; karen.gimenez@mail.ucv.es; 6 Research Group in Immunotherapy and Biomodels for Autoimmunity, Health Research Institute, Hospital Universitario y Politécnico La Fe, 46026 Valencia, Spain; lucas_barea@iislafe.es; 7 Department of Pathology, School of Medicine and Health Sciences, Universidad Católica de Valencia San Vicente Mártir, 46001 Valencia, Spain

**Keywords:** Myalgic Encephalomyelitis/Chronic Fatigue Syndrome (ME/CFS), Multiple Sclerosis (MS), human endogenous retrovirus (HERV), rituximab, autoimmunity, Epstein–Barr Virus (EBV)

## Abstract

This article summarizes the case of 30-year-old male diagnosed with Myalgic Encephalomyelitis/Chronic Fatigue Syndrome (ME/CFS) and its longitudinal follow-up, which provided a secondary diagnosis of Multiple Sclerosis (MS) eight years later. The most impactful result was his response to rituximab treatment after the systematic failure of prior treatments. Although the expression of endogenous retroviral proteins has been associated with autoimmunity, the patient did not show increased expression of the toxic protein HERV (human endogenous retrovirus)-W ENV, a target of the ongoing clinical trials with temelimab in MS and long COVID-19 cases. However, genome-wide HERV transcriptome analysis by high density microarrays clearly revealed a distinct profile in the patient’s blood supportive of an altered immune system. Limitations of the study include sub-optimal frequency of magnetic resonance imaging to monitor lesion progression, and similarly for reassessment of HERV profiles after rituximab. Overall, the coincidence of HERV alterations and the impactful response to rituximab presents the possibility of additional, more specific, therapeutic targets encoded by other HERV elements yet to be discovered.

## 1. Introduction

Myalgic Encephalomyelitis/Chronic Fatigue Syndrome (ME/CFS) is defined as a chronic disorder classified by the WHO as post-viral fatigue syndrome (ICD-11 8E49 code), while Multiple Sclerosis (MS) (ICD-11 8A40 code) is known as a chronic, inflammatory demyelinating disease of the central nervous system that includes three categories (relapsing/remitting, secondary progressive, and primary progressive) [1].

Diagnosis criteria for ME/CFS rely on clinical symptoms [2,3,4], as no validated biomarker exists, with post-exertional malaise or PEM playing a significant role, including fatigue, pain, and cognitive, intestinal, and sleep disturbances, among others, limiting a patient’s daily performance in mildly affected patients and driving bed confinement in severe cases. By contrast, MS is a complex neurological disorder characterized by inflammation in the central nervous system (CNS), leading primarily to demyelinating tissue damage. It affects approximately 2.8 million people worldwide [5]. MS represents a primary chronic, non-traumatic neurological disorder affecting young individuals with a high impact on patients’ quality of life [6]. The predominant presentation of MS manifests as relapsing episodes—relapsing MS (RMS) [7]. Clinical relapses lead to acute neurological damage, characterized by varied clinical manifestations and subsequent recovery. Natural history studies indicate that close to 40% of these patients undergo steady neurological deterioration without evident relapses, transitioning into a progressive phase of the disease—progressive MS (PMS) [8,9,10].

While no directional treatment exists for ME/CFS, being often multimedicated to palliate patients’ symptoms [11], there is a wide range of disease-modifying therapies available for MS, spanning from low- to high-efficacy options. These therapies enable neurologists to tailor treatment strategies based on individual prognostic factors [12,13]. In recent years, there has been a shift toward initiating treatment with high-efficacy disease-modifying therapies (DMTs) early in the disease course, as accumulating evidence suggests superior long-term outcomes [14,15,16,17,18,19,20]. Among these therapies, anti-CD20 monoclonal antibodies (e.g., rituximab) have emerged as a key approach to treat MS, specifically targeting B cell populations to modulate disease activity, as recently reviewed by Carlson et al. [21]. The rituximab mechanism of action relies on the activation of the complement system upon binding to CD20, a transmembrane protein present on pre-B and mature B lymphocytes, or by recruiting immune cells, such as macrophages and natural killer cells, to attack and destroy B cells, offering a benefit to treat B cell lymphomas, post-transplant lymphoproliferative disease, or autoimmunity [21,22].

This case report describes the longitudinal follow-up of a patient diagnosed with ME/CFS who developed central nervous system lesions compatible with a diagnosis of MS, the medication received, and the patient’s response to treatments.

While ME/CFS and MS can coexist, the remarkable phenotypic and neuroimmune overlaps between ME/CFS and MS may pose a challenge for a clearcut differential diagnosis [23]. Some observations are occasionally helpful; for example, extreme fatigue, appearing in both ME/CFS and MS, can occur independently of physical or mental exertion or be linked to neurological dysfunctions in MS, while ME/CFS fatigue is often triggered by exertion and does not improve with rest [23,24,25,26].

It is known that Epstein–Barr Virus (EBV) infection constitutes a risk factor for the development of MS [27], that the toxic HERV-encoded protein (HERV-W ENV) is often found overexpressed in MS [28], and that viral infections trigger the activation of HERV [29]. However, a mechanistic gap in knowledge to define precise HERV loci participation exists. In an effort to understand the potential mechanisms behind the patient’s diagnosis and response to rituximab, we evaluated the potential association of his diagnosis with derangement of peripheral blood mononuclear cells (PBMC) human endogenous retrovirus (HERV) expression, using as a reference previously reported healthy and ME/CFS HERV profiles [30].

To the best of our knowledge, this case study is the first to identify differences in HERV expression, at the loci level, in an EBV-positive male co-diagnosed with ME/CFS and MS who responded to rituximab, compared to healthy subjects and ME/CFS females. The potential mechanistic involvement of these HERV expression differences in the triggering or maintenance of disease, and/or the response to therapy, seem to justify the pertinence of future assessments.

## 2. Case Description

We present here the case of a male in his 30s diagnosed with ME/CFS and MS who experienced a significant improvement in his fatigue following initiation of treatment with rituximab.

The patient’s symptoms began in 2008, at age 18, characterized by episodic dizziness, malaise, blurred vision, weakness, and mental fatigue, with no identifiable trigger. Additionally, he had sustained sport injury fractures in his right/left 5th metacarpal and his clavicle earlier that year, followed by a bimalleolar ankle fracture in 2010, at age 20. Throughout this period, cognitive fatigue was his predominant symptom, significantly impacting his daily life. He experienced difficulties in concentrating, understanding, or solving problems, which forced him to change his academic pursuits towards less demanding studies. While physical fatigue occasionally led to episodes of being bedridden, he managed to maintain a relatively normal level of physical activity in the periods between extreme fatigue episodes. Dizziness and weakness with standing did not meet the criteria for postural orthostatic tachycardia or neuronally mediated hypotension. Ehlers Danlos was discarded by the absence of hyperlaxity or capillary fragility.

Despite evaluations by various specialists in the public health system, including internal medicine, neurology, rheumatology, psychiatry, and digestive medicine, no abnormalities were detected in clinical tests, leading to a referral to mental health services, where depression or anxiety was ruled out. In 2014, at age 24, his symptoms worsened when he began working in a physically demanding job, leading to new symptoms such as headache, fainting, tinnitus, dyspnea, difficulty speaking, abdominal pain, pain in the hands, neurovegetative crises during sleep, intolerance to temperature changes, and orthostatic intolerance. This resulted in prolonged sick leaves, with the patient living mostly confined to bed.

Further evaluations ruled out autoimmune, thyroid, myasthenia, endocrine, vitamin deficiency, and infectious pathologies, except for EBV IgG positivity in 2015, at age 25. Magnetic resonance imaging (MRI) in 2016, at age 26, revealed a small demyelinating juxtacortical lesion in the left temporal pole, but other investigations were unremarkable, leading to a diagnosis of ME/CFS. The patient met the 2011 International Consensus Criteria [2], as well as Canadian [3] and IOM (Institute of Medicine) 2015 criteria [4]. Prescribed supplements, including magnesium, NADH, D-ribose, L-carnitine, ubiquinol, melatonin, vitamin B1, alpha-lipoic acid, vitamin D, and LDN (Low-Dose Naltrexone), failed to improve his symptoms. Supplements were prescribed, not because there were evident deficiencies, but on the basis that they could help improve the patient’s fatigue.

**Figure 1:**
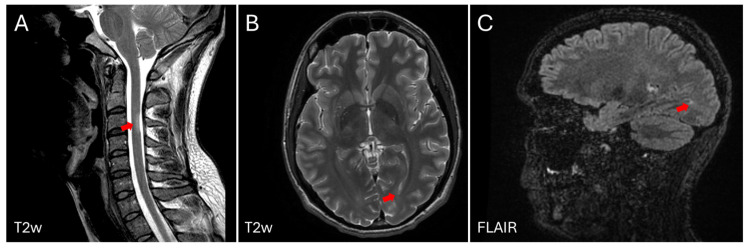
Magnetic resonance imaging (MRI) of the brain and spinal cord of the patient described in the clinical case, at age 31. Two subtle lesions can be observed in T2 sequences of the cervical spinal cord at the level of C3, as indicated with a red arrow (**A**) and in the white matter (WM) of the brain at the level of the temporal horn of the left lateral ventricle, pointed with a red arrow (**B**). Image obtained in FLAIR sequence, which enhances the lesions in WM (**C**), as indicated with a red arrow. The images were obtained in a 3-Tesla MRI (General Electric, Signa HDx, Boston, MA, USA).

In June 2017, at age 27, he decided to stop all treatments, and in July 2017, he experienced sensory loss in his legs with a sensory spinal cord level from C6 to D2, prompting a referral to the Neurology Department. Electromyoneurography (EMG) was normal, and a lumbar puncture revealed intrathecal production of antibodies (oligoclonal bands) not present in serum (IgG and IgM). Evoked sensory potentials were altered on both sides. During the diagnostic panel, anti-Acetylcholine receptor (AchR) antibodies were positive, although this finding was of unknown significance with the normal EMG. Brain and spinal cord MRIs revealed a new demyelinating lesion at the spinal cord level, fulfilling spatial and temporal dissemination criteria for MS diagnosis according to McDonald criteria 2017 [31], with two foci in the medulla and parietal lobe (Figure 1).

He was initially prescribed oral teriflunomide, a low-efficacy DMT [12], which was discontinued due to adverse effects, including tachycardia and tiredness. Subsequently, a switch to subcutaneous glatiramer acetate (GA) was tried for 3 years. Although the disease remained stable, there was no symptomatic improvement, and therefore it was decided to discontinue it. To address fatigue symptoms, amantadine, modafinil, and fampidrine [32] were trialed but proved ineffective. Amitriptyline was added for paresthesic symptoms at night, along with lormetazepam to improve sleep disturbances. Although well tolerated in general, several episodes of immediate post-injection reaction were experienced by the patient, characterized by shortness of breath and palpitations for 15 min, which became uncomfortable due to their increased frequency.

Following a new relapse of disease activity for MS, at the age of 31, with evidence of radiological inflammation in the MRI (Figure 1), rituximab, an anti-CD20 agent aimed at eliminating the B cell population of lymphocytes, was prescribed in December 2021.

The treatment regime consisted of an initial dose of 1 g on days 1 and 15 of the first month, followed by nine monthly doses of 1 g, adjusted based on the repopulation of B cells. This treatment resulted in a significant improvement in fatigue after the third dose, enabling increased mobility and participation in daily activities such as walking and dining out. Prior to treatment, the patient’s condition was as follows:

1. Physically, he was unable to leave the house (only for medical appointments, with significant subsequent deterioration, resulting in an increase in symptoms and increased fatigue requiring continuous rest). He was bedridden most of the day. He was able to perform personal self-care: grooming, dressing, eating at the table, and showering (he had to adapt his bathroom settings and did not do so daily).
2. Cognitively, his computer activity ranged from 10 min to a maximum of one hour a day on days when he felt better.
3. He suffered from a weekly migraine that could last up to three days.
4. He experienced daily symptoms of moderate to severe intensity: dizziness upon standing, feeling immediately tired and unsteady, concentration problems, memory problems, palpitations, and muscle pain.
5. Severe photosensitivity that impaired his vision.

After treatment, the patient’s condition improved at different levels:

1. Physically, he could leave the house 2–3 times a week and take gentle walks for an hour. He could help around the house with simple tasks such as cooking or cleaning, although he could not fully perform these tasks.
2. Cognitively, he could use the computer daily, without the deterioration that used to be triggered, but never for more than an hour at a time.
3. He reported a migraine once a month.
4. The rest of the symptoms persisted, but at a mild to moderate level. He tolerated standing for longer. For muscle pain, he is receiving treatment from a home physiotherapist, and it has almost completely resolved this issue.
5. Photosensitivity persists, and he still requires sunglasses, but it does not impair his vision.

He has not experienced any changes in his bowel movements before or after treatment; he has chronic constipation and requires laxatives.

He did not report infections or allergies.

On the Bell scale for severity assessment, the patient changed from a level of 10 to 20, meaning he doubled his previous capacities. The patient’s neurological condition remains stable, with fatigue stabilized.

EBV, as well as other viral infections unleashes the otherwise silenced expression of human endogenous retrovirus (HERV) [29], potentially leading to sterile inflammation and additional symptoms that resemble those of acute exogenous infections. In particular, overexpression of the toxic HERV-W ENV protein has been detected in MS patients [33], as well as in ME/CFS and post-COVID conditions [34], indicating a relationship of this protein with post-viral disease chronicity.

**Figure 2:**
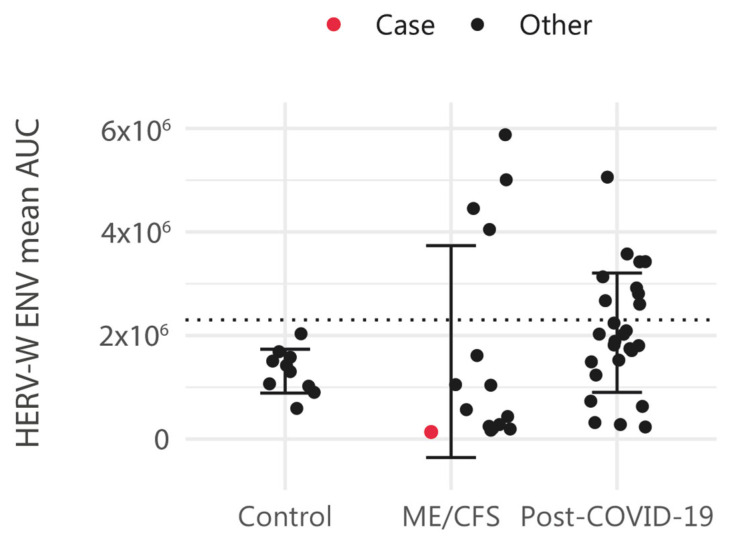
HERV-W envelope (ENV) antigenemia in control cases (n = 10), ME/CFS (n = 15) and post-COVID-19 condition subjects (n = 26). Levels of HERV-W ENV protein in plasma are shown as the mean area under the curve (AUC), as formerly described [34]. HERV-W ENV protein level in plasma is highlighted in blue for the study case. The dotted horizontal line indicates the cutoff minimum value to be considered positive.

To determine whether HERV-W ENV is overexpressed in the plasma of this patient, who was positive for EBV IgGs in 2015, we assessed HERV-W ENV protein levels following the protocol previously described in [34] and compared it to those in other ME/CFS cases and control samples (Figure 2) (Supplementary Table S1) (patient’s archived sample National Registry in Biobanking collection C0006024 obtained in 2016). The results showed that the mean HERV-W ENV level of this patient’s was 1.36 × 10^5^ area under the curve (AUC), which was below the positivity cutoff value (mean AUC: 2.30 × 10^6^), defined as the mean + 2 standard deviations of control samples.

**Figure 3:**
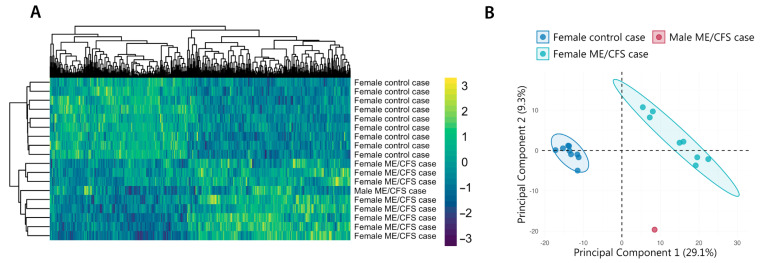
HERV clustering (**A**) and principal component analysis (**B**) of the ME/CFS male case reported in this study, along with 8 female ME/CFS cases and 9 healthy volunteer samples. Analysis includes all HERV probes of custom HERV-V3 arrays [36], displaying significant differential expression between ME/CFS and healthy control groups (FDR < 0.1 and |log2FC| > 1). Clustering heatmap was built with pheatmap [37] and PCA with the FactoMineR package [38].

To further explore potential HERV alterations in this patient, we performed a genome-wide analysis using high-density microarrays, as described in [30]. The results of this study case were compared to those of other age- and BMI-matched ME/CFS cases and healthy volunteers. Bioinformatic analysis, conducted with the R “limma” package [30,35], identified 502 differentially expressed HERV probe sets between ME/CFS patients and healthy controls (*p*-value < 0.1 and absolute log2 fold-change > 1) (Supplementary Table S2). Hierarchical clustering of HERV expression profiles revealed that the patient’s sample clustered with other ME/CFS cases, despite being from a male subject (Figure 3A). To further investigate the patient’s clustering pattern, we conducted principal component analysis (PCA). This analysis revealed that while the patient’s sample did not perfectly align with female ME/CFS cases (Principal Component 2, 9.3%), his HERV profile was closer to ME/CFS patients’ than to control’s (Principal Component 1, 29.1%) (Figure 3B), indicating potential epigenetic alterations in his immune cells that despite differing from both comparison groups aligns with that of ME/CFS cases.

To understand the potential cell role of the top overexpressed HERVs in the study case, we first selected those with the highest expression levels in the study case sample with respect to the rest (control or ME/CFS case), which turned out to be 29 out of the 502 differentially expressed (about 6% of them) (Supplementary Table S3), and then searched for their closest annotated genes. Gene names, genomic distances to HERVs with the highest expression in the study case, and their position within the matched gene were determined using the Goldmine package in RStudio (v4.4.0) [39] (Table 1). The data show that of the 29 HERVs allocated, 10 (34.5%) corresponded with intergenic regions less than 100,000 bp away, while the remaining 19 (65.5%) seemed to be encoded within genes. From the latter, most (thirteen) were encoded within introns (68.4%), four lay within promoter regions (21%), one lay within the 3’UTR region, and one lay within a coding exon (5.3% each). In addition to regulatory lncRNAs and novel transcripts, hit genes were found to be associated with development, infection, metabolism, and neural homeostasis; for example, *QKI* was involved in myelinization and oligodendrocyte differentiation, and *LINGO2* had a role in the glutamatergic synapse.

The high-density HERV-V3 custom microarray used to evaluate HERV expression levels at the loci level (174,852 HERV elements) in this patient also contains probes to measure 1559 genes involved in immunity, inflammation, cancer, central nervous system affections, differentiation, and telomere maintenance [30,36], as well as a series of probe sets to detect the presence of 289 exogenous active infections including dsDNA viruses, RT viruses, and ssDNA viruses [36]. However, all values obtained to assess genes and virus sequences in this patient did not reach levels above mean values of the group of ME/CFS female patients used as a reference, indicating an absence of marked inflammation, or active exogenous infections, or virus reactivation.

## 3. Discussion

This case report informs of a patient having received an initial diagnosis of ME/CFS (2015), at age 25, and a later diagnosis of MS (2017), at age 27, who, having shown a lack of response to several treatments for the following three years, then showed a striking response to rituximab in 2020, at age 30, continuing in remission until present. Although MS relapse frequency is highly variable, as is the case for factors affecting MS-associated fatigue [40], recent meta-analysis reports a reduction in MS relapses with rituximab treatment [41]. Clinical trials assessing the potential benefit of ME/CFS with rituximab are more limited, with an initial partial success in the reduction in symptoms [42] and failure to show benefits by a continuation trial [43]. Discrepancies across trials may be associated with patient heterogeneity, the medical regime applied, or other factors. In this case report, the patient not only showed clear improvement after only three doses of treatment with rituximab, but also his neurological condition remains stable, with reduced fatigue now for some years after the applied 9-month treatment regime. The limited external validity of single study cases and therefore the restricted applicability of these results should be stressed at this point.

The patient displayed auto-antibodies against AchR (AchR-aAbs), which is a typical finding in myasthenia gravis (MG), an autoimmune disease displaying impaired neuromuscular junction transmission with about 85% of patients presenting AchR-aAbs [44]. Thus, a mechanism mediated by AchR-aAbs contributing to patient improvement by rituximab therapy cannot be discarded [45]. However, there are no conclusive studies on the predictive value of these antibodies in asymptomatic patients or those with normal EMG [46]. The patient’s levels were moderately positive, suggesting possible autoimmune disease, but were evaluated in the clinical context. Whether the successful outcome for this patient may be due to other additional factors is presently unknown.

The lack of overexpression of the toxic HERV-W ENV protein, also known as Multiple Sclerosis Retrovirus (MSRV), which is frequently reported in MS patients [47] but also reported in some ME/CFS patients [33], was unexpected. Targeting MSRV with the monoclonal antibody temelimab is being studied [47]. On the other hand, our mechanistic findings on differential HERV profiles appear limited by the lack of comparison of HERV profiles of this case to male ME/CFS and MS cases. In fact, the literature reports molecular differences in the immune systems of male and female ME/CFS patients [48,49,50,51]. Whether molecular sex differences in ME/CFS include HERV profiles remains unknown at present. Nevertheless, the results of this patient’s HERV profile seem to align better with other ME/CFS cases, despite the absence of sex matching, than with profiles of the healthy participants, in support of his ME/CFS diagnosis. In addition, the findings fit with those of previous reports of EBV infections leading to important endogenous retroviral repression in CD19 + B cells [52]. Despite an important downregulation of HERV expression in the PBMC of our patient, we observed prominent overexpression (overexpressed above all other reference samples; n = 17) (Supplementary Table S3) in about 6% of the differentially expressed HERVs. Interestingly, associated gene functions match with potential alterations of the patient’s physiology (Table 1). Although it is not possible to attribute our findings to CD19+ B cell epigenetic alterations or to any other PBMC subpopulations, the observed response of the patient to rituximab therapy suggests this may be a mechanism for EBV disease triggering worth exploring. Future mechanistic assessments of the response to rituximab may benefit from longitudinal HERV profiling.

EBV infections seem to constitute a causal factor for developing MS [53], as found for EBNA1-peptide epitope mimicry mechanisms, with the central nervous system protein glial cell adhesion molecule (GlialCAM) leading to nervous tissue lesions [54]. Similarly, mechanisms leading to autoimmune encephalitis after herpes simplex infections [55] or to other types of autoimmunity by still-unknown mediators (unknown foreign antigens) [56] have been described. Additional auto-antigen-related viral sequences will possibly be discovered in the years to come. In this respect, WHO’s definition of ME/CFS as a post-viral chronic disease and the rich bibliography relating ME/CFS to EBV, herpes, or other infectious agents [57,58,59], in addition to the presentation of symptom overlaps between ME/CFS, MS, and MG, including muscle weakness, sleep and sensory disturbances, or pain [60,61,62], highlights the pertinence of co-exploring the mechanisms underlying these diseases. It must be highlighted that despite the positive EBV serology in 2015, no markers of EBV reactivation were detected in the patient by microarray screening. The recently reported overexpression of TTMV9 transcripts in the female ME/CFS group used here as a reference [63], potentially indicating a weak immune system, was also absent in this patient.

The case reported here appears singular from the angle that the patient presents with features constituted by three different diseases, with a preceding diagnosis of ME/CFS supporting at least co-occurrence of ME/CFS and MS. Further work towards defining differential markers across these and other symptom-related diseases, thus promoting the development of targeted therapeutics, is urgently needed.

## 4. Conclusions

In summary, we present a male case with a co-diagnosis of ME/CFS and MS, with a positive EBV serology, also expressing AchR-aAbs, and showing multiple symptomatology and lesions in the spine and brain, who positively responded to a 9-month treatment regime of rituximab doses of 1 g and stayed in remission for at least 3 years after treatment. Despite no evidence of HERV-W ENV overexpression, as expected, the patient’s PBMC HERV profile was clearly in closer proximity to other ME/CFS profiles than with those of healthy participants, with potential sex discrepancies and/or connections to MS and MG; confirmation is pending. Derangement of HERV expression in B cells contained within the explored PBMC appears as a potential mechanistic explanation for the response of this patient to rituximab, and this is seemingly worth exploring in future cases. In addition, the possibility of individual responses to viral infections triggering the development of complex chronic states deserves further assessment.

## Figures and Tables

**Table 1:** Genes overlapping or proximal to HERVs showing the highest expression in the case report sample.

HERV Element	Gene	Alias	Description	Distance (bp)	Subregion
ERVL_8q11.23	*RB1CC1*	RB1 Inducible Coiled-Coil 1	The protein encoded by this gene interacts with signaling pathways to coordinately regulate cell growth, cell proliferation, apoptosis, autophagy, and cell migration.	0	intron
MST_5p14.1	*PURPL*	P53 Upregulated Regulator Of P53 Levels	lncRNA	0	intron
MLT1_Xq25	*TENM1*	Teneurin Transmembrane Protein 1	Involved in neural development, regulating the establishment of proper connectivity within the nervous system.	0	intron
MLT1_11q23.3	*PHLDB1*	Pleckstrin Homology Like Domain Family B Member 1	Involved in regulation of embryonic development; regulation of epithelial to mesenchymal transition; and regulation of microtubule cytoskeleton organization. Located in several cellular components, including basal cortex; cytosol; and intercellular bridge.	0	exon
MLT1_11p15.2	*SOX6*	SRY-Box Transcription Factor 6	The encoded protein is a transcriptional activator that is required for normal development of the central nervous system, chondrogenesis, and maintenance of cardiac and skeletal muscle cells.	0	intron
MLT1_4p16.3	*PCGF3*	Polycomb Group Ring Finger 3	Component of a Polycomb group (PcG) multiprotein PRC1-like complex, a complex class required to maintain the transcriptionally repressive state of many genes, including Hox genes, throughout development	0	intron
MLT1_8q24.21	*CYRIB*	CYFIP Related Rac1 Interactor B	Involved in several processes, including cellular response to molecule of bacterial origin; negative regulation of small GTPase-mediated signal transduction; and regulation of organelle organization. Located in mitochondrion.	0	intron
MLT1_9p21.1	*LINGO2*	Leucine Rich Repeat And Ig Domain Containing 2	Predicted to act upstream of or within positive regulation of synapse assembly. Predicted to be located in membrane. Predicted to be active in several cellular components, including extracellular space; glutamatergic synapse; and synaptic membrane.	0	intron
MER4_1q31.2	*Lnc-BRINP3-7*	Novel transcript	Novel transcript	0	promoter
ERV9_2q32.3	*CAVIN2-AS1*	CAVIN2 And TMEFF2 Antisense RNA 1	lncRNA	0	promoter
MER21_6q26	*QKI*	QKI, KH Domain Containing RNA Binding	The encoded protein is involved in myelinization and oligodendrocyte differentiation.	0	intron
LTR84_15q24.3	*LOC105370906*	Novel transcript	lncRNA	0	intron
ERV9_21q21.3	*MAP3K7CL*	MAP3K7 C-Terminal Like	Located in cytosol and nucleus.	0	promoter
MER21_22q12.1	*GRK3*	G Protein-Coupled Receptor Kinase 3	Specifically phosphorylates the agonist-occupied form of the beta-adrenergic and closely related receptors.	0	intron
MST_2q12.3	*LINC01789*	Long Intergenic Non-Protein Coding RNA 1789	lncRNA	0	intron
MLT1_6p11.2	*RAB23*	RAB23, Member RAS Oncogene Family	The encoded protein may play a role in central nervous system development by antagonizing sonic hedgehog signaling.	0	intron
THE1_12q21.31	*MGAT4C*	MGAT4 Family Member C	Predicted to enable acetylglucosaminyltransferase activity. Among its related pathways are Translation of Structural Proteins and Infectious disease.	0	3' end
MST_22q11.21	*USP18*	Ubiquitin Specific Peptidase 18	Among its related pathways are Toll Like Receptor 7/8 (TLR7/8) Cascade and Overview of interferons-mediated signaling pathway.	0	intron
MST_18q21.1	*MIR4527HG*	MIR4527 Host Gene	lncRNA	94	promoter
MLT1_4q35.1	*FAM149A*	amily With Sequence Similarity 149 Member A	An important paralog of this gene is *FAM149B1*.	3119	intergenic
MER61_11q12.1	*GLYAT*	Glycine-N-Acyltransferase	Mitochondrial acyltransferase, which transfers an acyl group to the N-terminus of glycine and glutamine, although much less efficiently.	7837	intergenic
HERV32_Xq13.1	*COX6CP12*	Cytochrome C Oxidase Subunit 6C Pseudogene 12	Pseudogene	10,819	intergenic
HERVL33_14q32.32	*ENSG00000289207*	Lnc-LBHD2-4	lncRNA	11,605	intergenic
MER61_8p12	*MTND5P41*	MT-ND5 Pseudogene 41	Pseudogene	12,771	intergenic
MLT1_6p12.1	*TINAG*	Tubulointerstitial Nephritis Antigen	This gene encodes a glycoprotein that is restricted within the kidney to the basement membranes underlying the epithelium of Bowman's capsule and proximal and distal tubules.	18,001	intergenic
LTR48_12q14.2	*LDHAL6CP*	Lactate Dehydrogenase A Like 6C	Pseudogene	19,473	intergenic
THE1_9q34.11	*Lnc-NTMT1-2*	Uncharacterized LOC105376292	lncRNA	30,032	intergenic
HERV16_1p34.3	*LINC01343*	Long Intergenic Non-Protein Coding RNA 1343	lncRNA	39,532	intergenic
MER4_18q23	*Lnc-PQLC1-5*	NONHSAG024230.2	lncRNA	71,187	intergenic

## Data Availability

The original contributions presented in this study are included within the article. Further inquiries can be directed to the corresponding author/s.

## Supplementary Materials

The supporting information can be downloaded at: https://www.mdpi.com/article/10.3390/ijms26104885/s1.

## Abbreviations

The following abbreviations are used in this manuscript:

ME/CFS	Myalgic Encephalomyelitis/ Chronic Fatigue Syndrome
MS	Multiple Sclerosis
MG	Myasthenia gravis
HERV	Human endogenous retrovirus
HERV-W ENV	Human Endogenous Retrovirus type W (tryptophan), Envelope protein
MSRV	Multiple Sclerosis Retrovirus
PBMC	Peripheral Blood Mononuclear Cells
EBV	Epstein–Barr Virus
DMT	Disease-modifying therapy
AUC	Area under the curve
AchR	Acetylcholine receptor
aAb	Autoantibody
